# PAQR4: A Critical Senescence-Related Gene Influencing Immune Evasion and Metastasis in Bladder Urothelial Carcinoma

**DOI:** 10.1155/humu/2227219

**Published:** 2025-09-12

**Authors:** Yizhang Sun, Xincheng Yi, Chenchao Zhou, Yuhua Huang

**Affiliations:** Department of Urology, First Affiliated Hospital of Soochow University, Suzhou, Jiangsu, China

**Keywords:** biomarker, bladder urothelial carcinoma, cellular senescence, immune microenvironment, immunotherapy, PAQR4

## Abstract

**Background:** Bladder urothelial carcinoma (BLCA) is a prevalent malignant tumor known for its high recurrence rates and limited therapeutic efficacy. Cellular senescence has been extensively shown to inhibit tumorigenesis via cell cycle arrest. Consequently, the identification of senescence-associated biomarkers is essential for enhancing the diagnosis, prognosis, and immunotherapeutic outcomes of BLCA.

**Method:** This study integrated the TCGA-BLCA and GSE13507 datasets to analyze senescence-related genes. ConsensusClusterPlus was employed for cluster analysis, while immune infiltration was assessed using CIBERSORT. A diagnostic and prognostic model for BLCA was developed and validated through the combination of various machine learning algorithms. Experimental validation was conducted using qRT-PCR, colony formation, and Transwell assays to evaluate the functional role of PAQR4.

**Result:** Our study revealed that aging-related samples demonstrated improved survival rates and a lower incidence of high-grade tumors. Cluster analysis identified two distinct subgroups of BLCA characterized by unique immune infiltration profiles and varying responses to immune checkpoint blockade. The diagnostic and prognostic models developed from aging-related genes were subsequently validated. Notably, PAQR4 was identified as a critical aging-related gene linked to poor prognosis and an immunosuppressive microenvironment. The knockdown of PAQR4 significantly inhibited the proliferation and metastasis of BLCA cells.

**Conclusion:** PAQR4 is a novel biomarker and therapeutic target for BLCA, influencing cellular senescence, immune evasion, and metastasis. This study provides insights into the senescence-related mechanisms and offers tools for precision diagnosis and the optimization of immunotherapy in BLCA.

## 1. Introduction

Bladder urothelial carcinoma (BLCA) is one of the most prevalent malignant tumors of the urinary system, ranking ninth globally in terms of incidence among malignant tumors, with a steadily increasing trend observed annually. As reported in Cancer Statistics, 2023, roughly 82,290 people receive a BLCA diagnosis each year in the United States, leading to an estimated 16,710 fatalities [1]. These statistics highlight BLCA as a continuing and major issue in public health. The risk factors for BLCA are closely linked to smoking and occupational exposure to chemical substances, such as dyes and rubber products [2, 3]. Despite continuous advancements in diagnostic and treatment technologies, the high recurrence rate and complexity of treatment for BLCA pose significant clinical challenges. For instance, patients with nonmuscle-invasive BLCA require repeated transurethral tumor resection and intravesical instillation therapy, while those with muscle-invasive BLCA necessitate radical cystectomy combined with urinary diversion, which significantly diminishes their quality of life [4, 5]. Furthermore, existing diagnostic methods, such as cystoscopic biopsy, are invasive and may lead to infections. Although noninvasive urinary biomarkers are increasingly utilized, their sensitivity and specificity remain inadequate. Therefore, there is an urgent need to develop more precise diagnostic and therapeutic biomarkers to enhance early screening, efficacy evaluation, and recurrence monitoring.

Cellular senescence is intricately involved in both the formation and advancement of tumors. Particularly in BLCA, senescence is recognized as a significant mechanism counteracting cancer. This phenomenon inhibits the proliferation of cancer cells by causing a permanent halt in the cell cycle, a complex process that is governed by the interplay of multiple genes and signaling pathways [6, 7]. Nevertheless, the impact of cellular senescence extends beyond merely suppressing tumors; the senescence-associated secretory phenotype (SASP) it releases can, under specific circumstances, enhance tumor advancement and metastasis [8, 9]. In the framework of BLCA, researchers have discovered a notable connection between cellular senescence and the efficacy of tumor immunotherapy. Investigations indicate that senescent cells may alter the tumor microenvironment through the secretion of various factors, thereby influencing the immune system's response [10, 11]. These components have the capacity to draw immune cells to recognize and remove senescent cells, which could contribute to a decrease in tumor formation and expansion. On the other hand, if the clearance of these senescent cells occurs later than required, it may result in an immune-suppressive environment that supports tumor advancement [12, 13]. A study utilizing a risk model derived from macrophage senescence-related genes can evaluate prognosis, immunotherapy sensitivity, and chemotherapy response in bladder cancer. Furthermore, another investigation indicated that cellular senescence may impede lymph node metastasis in bladder cancer by diminishing M2 macrophage infiltration [14, 15]. Moreover, the contribution of cellular senescence to cancer treatment has garnered considerable interest in recent years. Investigations are underway to explore the feasibility of employing senescence as a method to induce tumor cells for cancer therapies. This approach is viewed as a potential augmentation to conventional treatments, particularly in the context of certain anticancer agents, where cellular senescence may emerge as a desirable therapeutic result [16, 17]. Nonetheless, effectively harnessing the dual nature of cellular senescence presents challenges that necessitate additional investigation and analysis.

Cellular senescence is a complex biological process characterized by the activation of the P53 signaling pathway. P53 is an important tumor suppressor protein that plays a crucial role in the cellular response to DNA damage and other stressors. The activation of P53 is often accompanied by the upregulation of the cell cycle inhibitor p21, a process that can lead to cell cycle arrest and the induction of cellular senescence [18, 19]. During cellular senescence, the expression of the proliferation marker KI67 is typically suppressed. KI67 is a nuclear protein associated with cell proliferation, and its expression level significantly decreases when cells enter a senescent state. This suppression reflects a reduction in the proliferative capacity of senescent cells [20]. Furthermore, the activation of the P53 signaling pathway mediates cell cycle arrest not only through p21 but also influences the senescence process via other pathways. For instance, P53 can further promote cellular senescence by regulating the levels of intracellular reactive oxygen species (ROS) [21]. Studies have demonstrated that the activation of the P53 signaling pathway is closely linked to cellular senescence across various cell types. For example, in hepatocellular carcinoma, the activation of P53 can induce cellular senescence through the AMPK pathway [22]. In colorectal cancer, the loss of BRG1 promotes cellular senescence by affecting the SIRT1/P53/p21 signaling axis [23]. In summary, the activation of the P53 signaling pathway serves as a crucial marker of cellular senescence, while the suppression of KI67 indicates a decline in proliferative capacity. We identified senescence-associated samples in BLCA by analyzing the expression levels of P53 and KI67, which led to the discovery of differentially expressed genes. Utilizing machine learning methods in conjunction with experimental validation, we confirmed the critical role of PAQR4. Our findings contribute to the identification of novel immunotherapeutic targets and diagnostic markers for BLCA.

## 2. Materials and Methods

### 2.1. Datasets

This research employed two different datasets: TCGA-BLCA and GSE13507. The TCGA-BLCA dataset includes 406 samples of BLCA in addition to 19 samples from normal bladder tissue, while the GSE13507 dataset contains 165 BLCA samples and 9 samples representing normal bladder tissue.

### 2.2. Cluster Analysis

Cluster analysis was conducted utilizing ConsensusClusterPlus [24]. This involved agglomerative PAM clustering based on a 1-Pearson correlation distance metric, with 80% of the samples being resampled over 10 iterations. The empirical cumulative distribution function plot was employed to identify the optimal number of clusters.

### 2.3. Immune Infiltration

The immunedeconv R package aims to estimate the proportions of various immune cell types, providing a consistent interface for accessing multiple methods of immune deconvolution [25]. Notably, CIBERSORT is highlighted as a deconvolution algorithm that is specifically employed in the analysis of tumors and diseases related to the immune system, and it is part of the immunedeconv package. Our study evaluated the levels of immune infiltration in different sample groups using the CIBERSORT algorithm.

### 2.4. Construct Diagnostic and Prognostic Models

To develop a trustworthy and precise diagnostic and prognostic model for BLCA, we utilized a range of machine learning techniques with varying configurations. The training dataset was obtained from the TCGA-BLCA repository, whereas the validation process was carried out using the GSE13507 dataset. Each algorithmic configuration was assessed using the area under the curve (AUC) metric, with the combination that achieved the highest mean AUC recognized as the optimal model.

### 2.5. Quantitative Reverse Transcription PCR (qRT-PCR)

RNA extraction from cellular samples was conducted utilizing Trizol reagent, a standard method known for its efficiency in isolating RNA. Following this extraction process, complementary DNA (cDNA) was synthesized through reverse transcription, using the RevertAid FirstStrand cDNA Synthesis Kit. This kit is recognized for its reliability in converting RNA templates into cDNA, which is a crucial step for subsequent analysis. To quantify the cDNA, qRT-PCR was performed using the 7900HT Fast Real-Time PCR System manufactured by Applied Biosystems. The reaction setup for qRT-PCR was carefully executed according to the established protocols specified below, ensuring accurate and reliable results in the quantification of gene expression. Below are the sequences for the primer pairs targeting the genes: PAQR4 (Forward: TACCTGCACAACGAACTGGG, Reverse: AAGAGGTGATAGAGCACGGAG) and GAPDH (Forward: GAGCCACATCGCTCAGACAC, Reverse: GCCCAATACGACCAAATCC).

### 2.6. Cell Culture

The human bladder cancer cell lines UMUC3 and T24 were sourced from the Chinese Academy of Cell Collection located in Shanghai, China. These cell lines were cultured in RPMI 1640 medium, which was supplemented with 10% fetal bovine serum (FBS) and 100 U/mL of penicillin–streptomycin. The cultures were maintained at a temperature of 37°C in a humidified incubator with an atmosphere containing 5% carbon dioxide.

### 2.7. Colony Formation and Migration Assays

To facilitate colony formation, a total of 1000 transfected cells were carefully seeded into six-well plates and incubated in RPMI-1640 medium supplemented with 10% FBS. Following a period of 2 weeks, the established cell colonies were fixed in methanol for 15 min to preserve their structure, after which they were stained with a 0.1% solution of crystal violet (obtained from Sigma-Aldrich) for a duration of 30 min. Once the staining process was complete, the visible colonies were documented through photography, and their numbers were quantified using ImageJ software for accurate analysis. A cell migration assay was conducted utilizing a transwell chamber (Corning, United States) following established protocols [26]. Briefly, cells that had been transfected were suspended in 150 *μ*L of serum-free medium and introduced into the upper chamber of the transwell, while the lower chamber contained 600 *μ*L of medium enriched with 10% FBS. After incubating for 48 h, the upper chamber was cleared of cells using cotton wool, and those that migrated to the lower side were fixed with 4% paraformaldehyde and stained with 0.1% crystal violet (Beyotime). Finally, five random fields of view were captured using an inverted microscope, and the cells in each well were counted.

### 2.8. Wound Healing Assay

Inoculate the cells into a 6-well plate at a density of 4 × 10^5^ cells per well and maintain culture until they reach confluence. Use a sterile 200 *μ*L pipette tip to generate a consistent scratch across the cell monolayer. After washing with PBS to remove any detached cells and debris, incubate the cells at 37°C, capturing images of the scratch at 0, 24, and 48 h.

### 2.9. Statistical Analysis

Statistical analyses were conducted utilizing R language Version 4.2.0 in addition to GraphPad Prism 10, with *p* values deemed statistically significant if they were below 0.05.

## 3. Result

### 3.1. Identification of Senescence-Related Genes in BLCA

**Figure 1:**
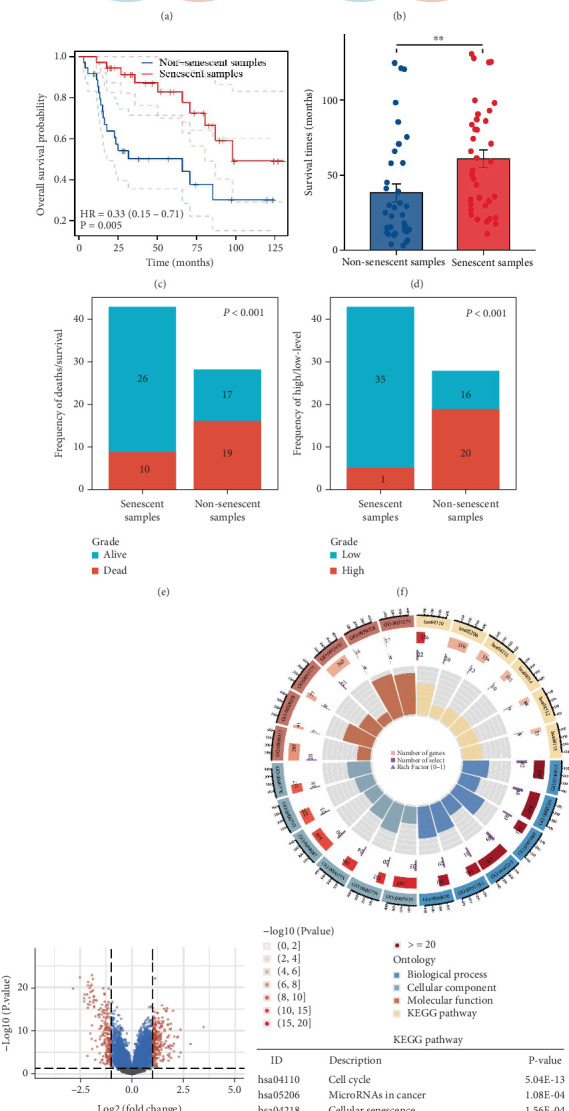
Differential analysis of senescence-related samples in BLCA. (a) Identification of senescent samples. (b) Identification of nonsenescent samples. (c) Survival analysis. (d) Survival time analysis. (e) Analysis of survival/death ratios. (f) Pathologic grading analysis. (g) Analysis of differences. (h) Enrichment analysis.

P53 operates as an initiator gene of cellular senescence and triggers senescence by regulating its downstream P21 [27]. The onset of cellular senescence commonly involves a decreased capacity for cell proliferation. MKI67, a frequently employed marker for proliferation, can be used to examine the proliferative activity of tumor cells. Therefore, we pooled P53 high-expression samples (above the median expression level of P53) and KI67 low-expression samples (below the median expression of KI67) as senescent samples, and P53 low-expression samples (below the median expression of P53) and KI67 high-expression samples (Above the median expression level of KI67) as nonsenescent samples in the GSE13507 dataset (Figure 1a,b). A survival analysis comparing senescent and nonsenescent samples revealed significantly higher survival rates in the former (Figure 1c). In addition, the group of cells that had undergone senescence demonstrated significantly better survival time and survival/death ratio compared to the nonsenescent group (Figure 1d,e). Moreover, the senescent group exhibited a significantly lower number of high-grade BLCAs in comparison to the nonsenescent group (Figure 1f). Finally, differential and enrichment analyses were conducted on aging and nonaging samples, using a threshold for screening differentially expressed genes defined as “*p* value <0.05 and fold change > 2 or fold change < −2.” The results indicated that the differentially expressed genes between the two groups were significantly associated with cellular senescence, the cell cycle, and the P53 signaling pathway (Figure 1g,h).

### 3.2. Cluster Analysis of Senescence-Related Genes

**Figure 2:**
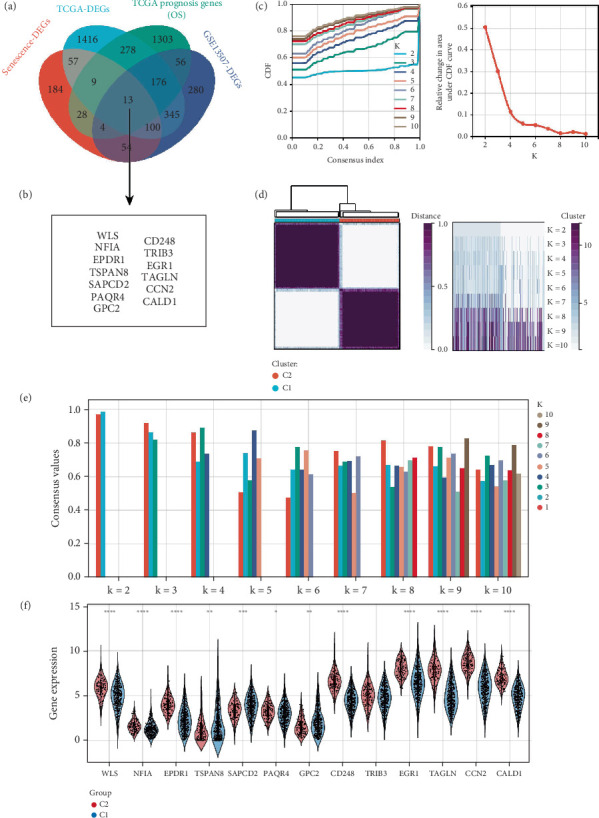
Cluster analysis divided the BLCA samples into 2 clusters. (a, b) Screening of differential prognostic genes related to senescence via Venn diagrams. (c) The CDF curves. (d) The CDF Delta area displays the CDF curve. (e) The sample distribution varies for different k values. (f) The differential expression of senescence-related genes.

Following a separate analysis of the senescent and nonsenescent cohorts, we included the TCGA-BLCA dataset in our research. In conclusion, we uncovered 13 differentially expressed genes related to senescence that showed a strong correlation with patient outcomes in both the TCGA-BLCA and GSE13507 datasets (Figure 2a,b). In recent times, the concept of precision medicine has focused on categorizing individual study participants into subgroups. A key illustration of this is the molecular typing of breast cancer, which showcases that different subgroups display unique pathogenic mechanisms and vary in clinical prognostic traits. Consequently, we performed subcomponent classification of the TCGA-BLCA dataset, which was distinguished by the expression of senescence-related genes (Figure 2c,d). We demonstrate the consistency of our sample clustering for each analysis with varying fractals, as depicted in Figure 2e. We obtained the optimal number of clusters, with a *K* value of 2. To ascertain the optimal quantity of clusters, the *K* value that produced the minimum “fuzzy clustering ratio” was identified, a method commonly employed in consensus clustering studies. The PAC index, which acts as an indicator of central tendency, is defined as the consensus index within the interval (*u*1, *u*2) ∈ [0, 1], where u1 approaches 0 and u2 approaches 1 (for instance, *u*1 = 0.2 and *u*2 = 0.8). A lower PAC score implies a smoother transition in the central segment, with reduced inconsistencies across the shuffled clustering attempts. The cumulative distribution curve, along with its area, revealed that the highest average consistency within the group occurred when *K* was set to 2. AKAP7, CDCA2, PRPF19, PRR11, and STIP1 did not show statistically significant differential expression in either Cluster 1 or Cluster 2, while the remaining senescence genes exhibited significant differential expression in both clusters.

### 3.3. Analysis of the Correlation Between Different Clusters and Immune Infiltration in BLCA

**Figure 3:**
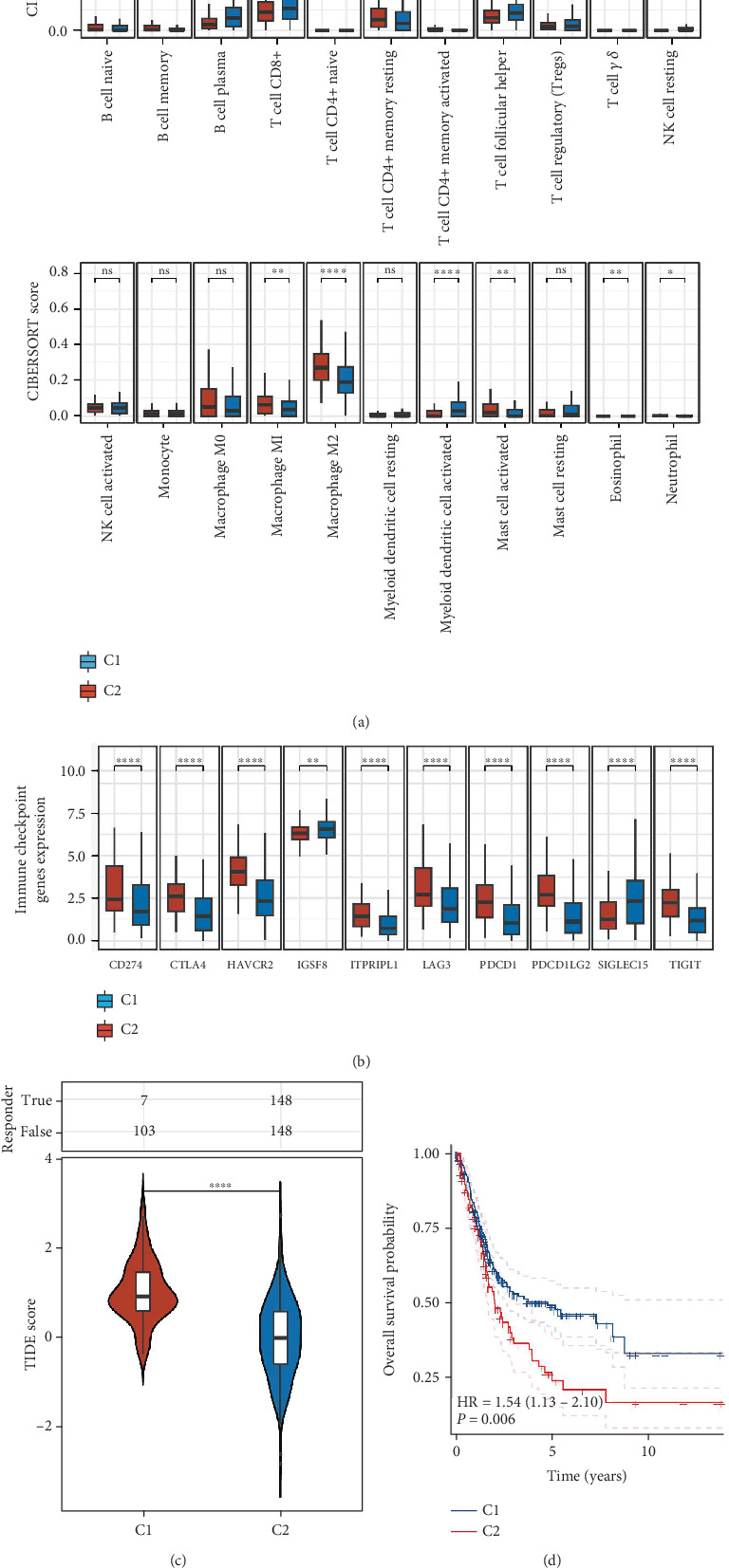
Analysis of the correlation between senescence related genes and immune infiltration. (a) Variation in the expression of immune infiltration-related cells between Cluster 1 and Cluster 2. (b) Comparison of immune checkpoint gene expression in Clusters 1 and 2. (c) The TIDE algorithm was utilized to predict potential responses to immune checkpoint blockade. (d) Analysis of survival outcomes in Cluster 1 versus Cluster 2.

Initially, we employed the CIBERSORT algorithm to examine the differences in the expression levels of immune infiltration-related cells between Cluster 1 and Cluster 2. Notably, there was a significant variation in the populations of B cell plasma, T cell CD4+ naive, T cell CD4+ memory resting, T cell CD4+ memory activated, T cell follicular helper, T cell regulatory (Tregs), resting NK cells, monocytes, M0 macrophages, M1 macrophages, M2 macrophages, activated myeloid dendritic cells, and neutrophils when comparing Clusters 1 and 2 (Figure 3a). We further examined the expression differences of immune checkpoint–related genes between Clusters 1 and 2, revealing that all checkpoint-related genes exhibited significant discrepancies (Figure 3b). The introduction of immune checkpoint blockade (ICB) therapy has transformed cancer treatment in humans, and we identified two clusters which may indicate responsiveness to immune checkpoint inhibitors, based on expression profiling data derived from the Tumor Immune Dysfunction and Exclusion (TIDE) algorithm. A high TIDE score corresponds to a reduced efficacy of ICB therapy and shorter survival post-ICB treatment. Consequently, Cluster 2 exhibits a lower efficacy with ICB and a decreased survival rate compared to Cluster 1 under ICB treatment (Figure 3c). Additionally, we assessed the survival differences between the two clusters and demonstrated that Cluster 2 had a significantly lower survival rate than Cluster 1 (Figure 3d). This result aligns with the previously mentioned treatment outcomes related to ICB.

### 3.4. Constructing Diagnostic and Prognostic Models

**Figure 4:**
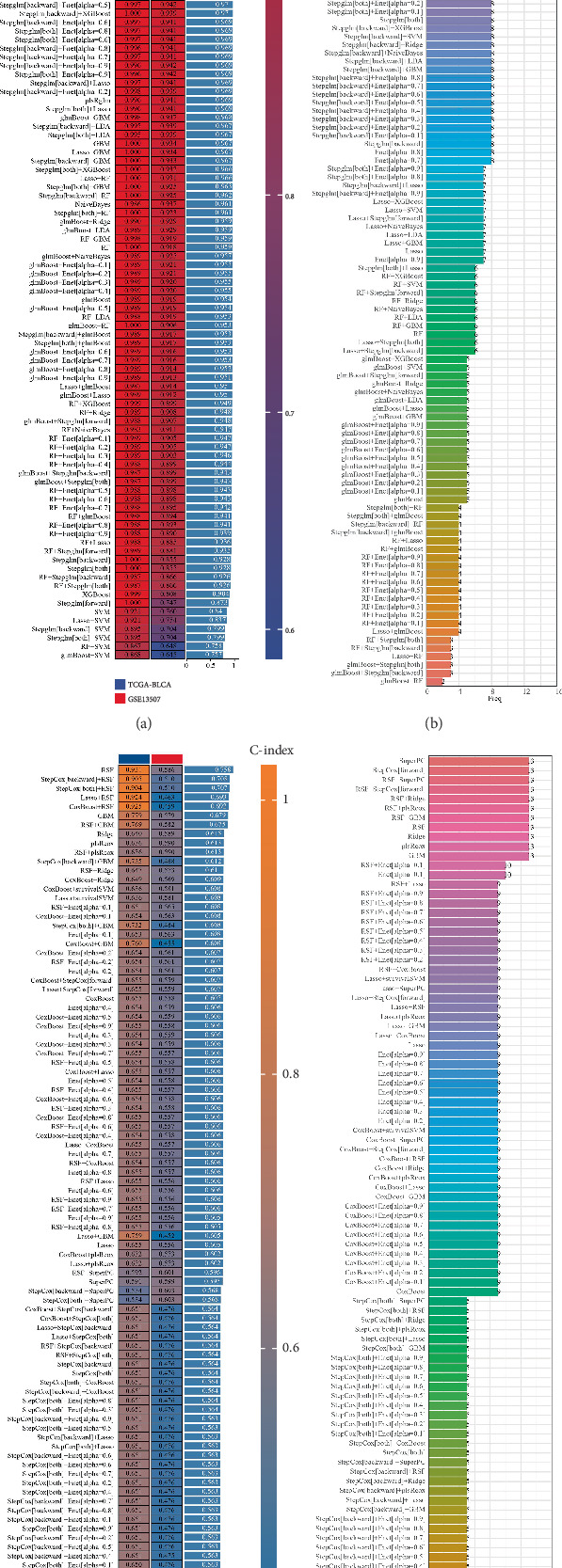
Constructing a cell senescence-related diagnostic and therapeutic model. (a) AUC values for diagnostic models created through the use of various algorithm combinations. (b) The quantity of genes integrated into diagnostic models designed with different combinations of algorithms. (c) AUC values for prognostic models established by utilizing various algorithm combinations. (d) The tally of genes included in prognostic models developed with different algorithm combinations.

To investigate the diagnostic and therapeutic relevance of genes associated with cell senescence in patients with BLCA, we incorporated data from the TCGA-LIHC and GSE13507 datasets into our study. Utilizing various algorithms, we first established a diagnostic model, which demonstrated that the Lasso + Stepglm [forward] algorithm had an outstanding predictive performance, with an average AUC of 0.976 across both datasets (Figure 4a). The prognostic model generated by this algorithm featured seven significant genes: NFIA, TSPAN8, PAQR4, GPC2, CD248, TRIB3, and EGR1 (Figure 4b). Following this, we created a separate prognostic model using the TCGA-LIHC and GSE13507 datasets. Our results suggested that the prognostic model established with the RSF algorithm was the most effective; nonetheless, the outcomes were only moderately satisfactory, yielding an average AUC of merely 0.758 across the two datasets (Figure 4c). This prognostic model encompassed all 13 genes evaluated in the analysis (Figure 4d).

### 3.5. PAQR4 as a Key Regulatory Gene in Cellular Senescence of BLCA Cells

**Figure 5:**
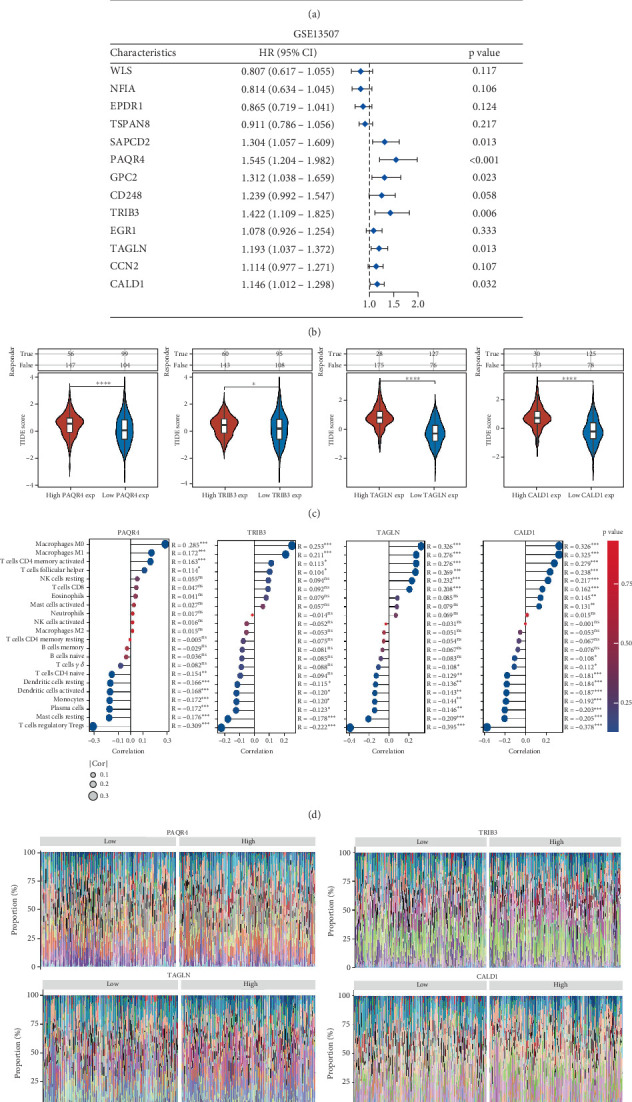
PAQR4 can serve as a target for immunotherapy in BLCA. (a) Analyzing the prognostic impact of genes associated with cellular senescence in the TCGA-BLCA dataset. (b) Evaluating the prognostic significance of cellular senescence-related genes in the GSE13507 dataset. (c) Examination of the relationship between genes linked to cellular senescence and immunotherapy outcomes in BLCA. (d, e) Examination of the relationship between genes linked to cellular senescence and levels of immune cell infiltration in BLCA.

To further identify the most critical genes among the 13 cellular senescence regulatory genes, we conducted a prognostic analysis using univariate COX regression. The results revealed that GPC2, PAQR4, TRIB3, TAGLN, and CALD1 exhibited significant prognostic differences. Notably, GPC2 displayed conflicting prognostic outcomes across the two datasets, prompting us to designate PAQR4, TRIB3, TAGLN, and CALD1 as the key cellular senescence-related prognostic genes for BLCA (Figure 5a,b). Furthermore, patients with low expression levels of PAQR4, TRIB3, TAGLN, and CALD1 showed improved outcomes when treated with immune checkpoint inhibitors (Figure 5c). Finally, we investigated the relationship between specific genes and the infiltration of immune cells in BLCA. The results of our analysis revealed that all four genes demonstrated a positive correlation with the levels of infiltration of several immune cell types, including M0 macrophages, M1 macrophages, activated CD4 memory T cells, and follicular helper T cells. Conversely, these genes showed a negative correlation with the infiltration levels of other immune cells, such as resting dendritic cells, activated dendritic cells, monocytes, plasma cells, resting mast cells, and regulatory T cells, as illustrated in Figures 5d,e. This dual nature of correlation emphasizes the complex interplay between these genes and the immune landscape within the tumor microenvironment (Figure 5d,e).

### 3.6. Functional Analysis of PAQR4 in BLCA

**Figure 6:**
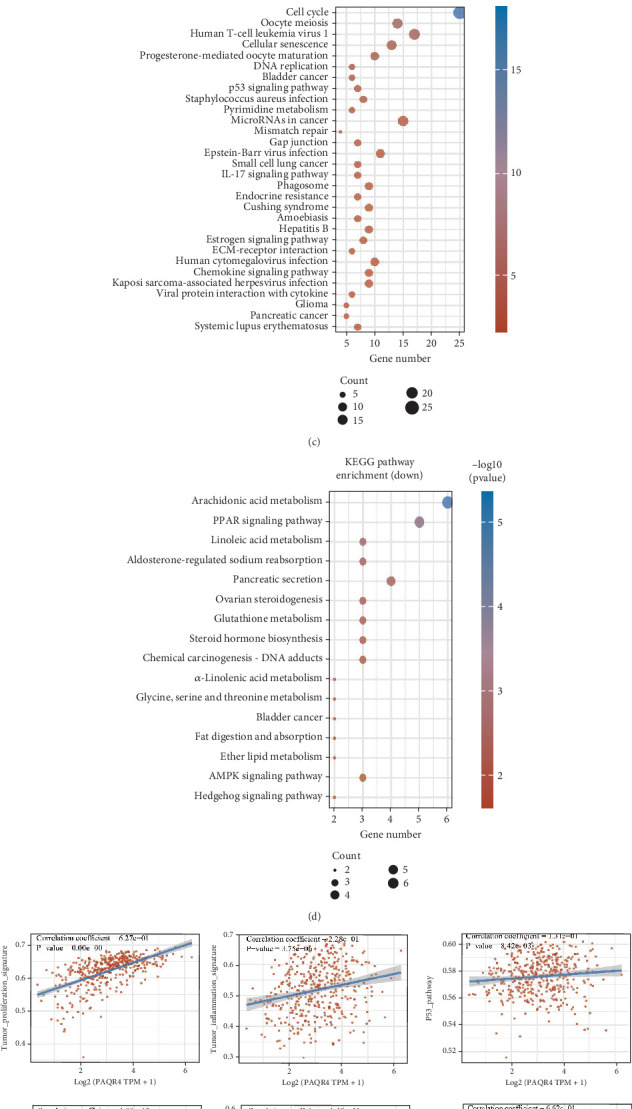
Functional analysis of PAQR4. (a, b) Difference analysis after grouping based on PAQR4 expression. (c, d) Analysis of PAQR4 functions by KEGG. (e) Analyzing the function of PAQR4 based on the ssGSEA algorithm.

In our previous analysis, we recognized PAQR4 as a crucial gene linked to senescence. As a result, we organized the TCGA-BLCA dataset according to the median expression level of PAQR4, labeling the group that fell below the median as the low-expression PAQR4 group and the group that exceeded the median as the high-expression PAQR4 group, subsequently conducting a differential analysis (Figure 6a,b). This analysis yielded 314 upregulated genes and 113 downregulated genes. Based on these differentially expressed genes, KEGG analysis indicated that PAQR4 in BLCA is significantly associated with pathways related to the cell cycle, cellular senescence, p53 signaling pathway, arachidonic acid metabolism, PPAR signaling pathway, and linoleic acid metabolism (Figure 6c,d). Finally, we also examined the correlation between PAQR4 and the tumor proliferation signature, tumor inflammation signature, p53 pathway, apoptosis, EMT markers, and angiogenesis using the ssGSEA algorithm, with results demonstrating a positive correlation among these factors (Figure 6e).

### 3.7. Knockdown of PAQR4 Inhibits BLCA Cell Proliferation and Metastasis

**Figure 7:**
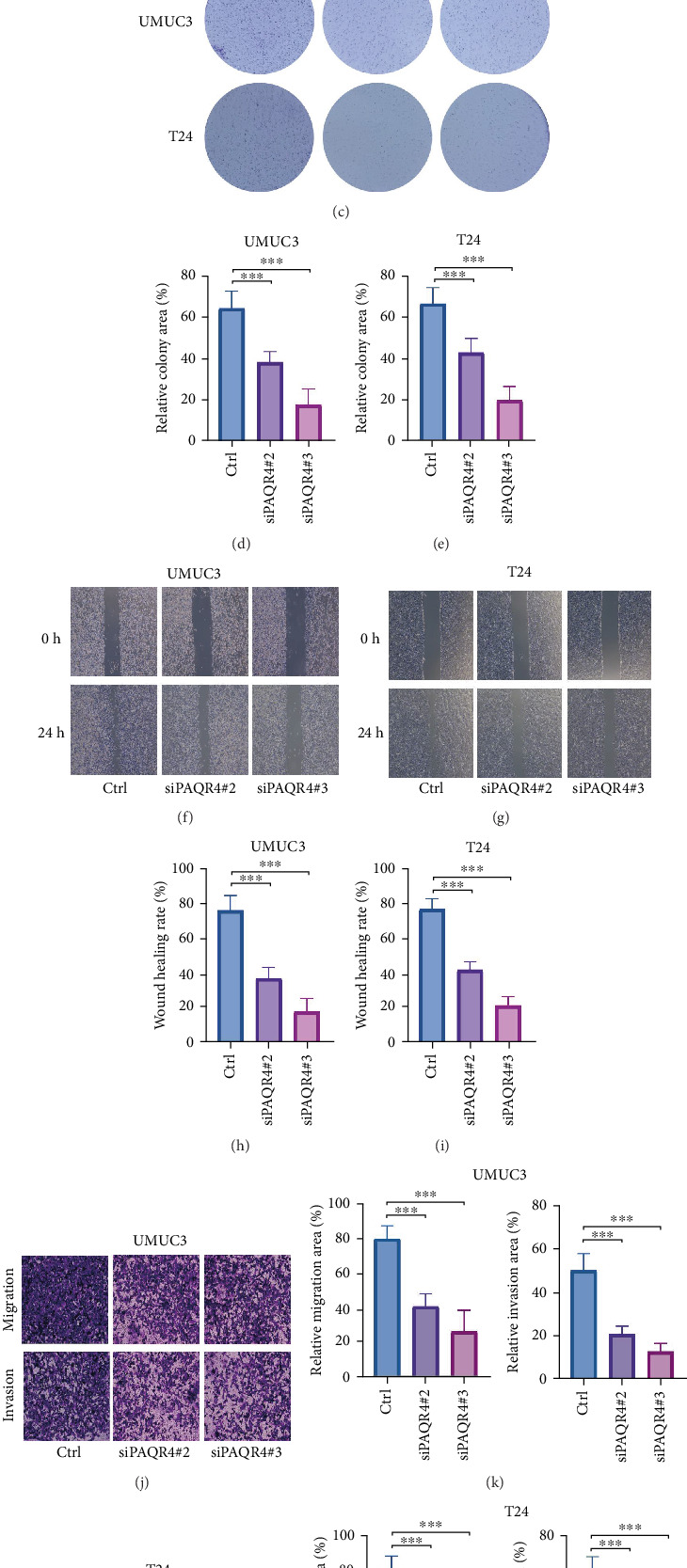
Detection of PAQR4's impact on the proliferation and metastatic ability of BLCA cells. (a, b) To evaluate the effectiveness of PAQR4 siRNA knockdown, qRT-PCR analyses were performed. (c–e) The assay for colony formation was carried out to assess the impact of PAQR4 reduction on cell proliferation. (f–i) A wound healing assay was executed to explore how PAQR4 knockdown affects cellular migration. (j–m) The influence of PAQR4 silencing on cell migration was analyzed using Transwell assays.

We assessed the effectiveness of various PAQR4 siRNA target sites through the qRT-PCR technique. Our findings revealed that si PAQR4 #2 and si PAQR4#3 displayed the strongest inhibitory effects (Figure 7a,b). As a result, these two target sites were employed for further cellular experiments. In the UMUC3 and T24 cell lines, we found that the depletion of PAQR4 notably suppressed cell proliferation (Figures 7c, 7d, and 7e). Furthermore, both scratch assays and Transwell experiments indicated that the reduction of PAQR4 significantly diminished the migration and invasion abilities of UMUC3 and T24 cells (Figures 7f, 7g, 7h, 7i, 7j, 7k, 7l, and 7m).

## 4. Discussion

BLCA represents a malignant tumor that primarily arises from the epithelium of the bladder. Presently, the primary methods of treatment for BLCA include surgical procedures and chemotherapy [28]. Despite significant advancements in these therapeutic strategies, the overall survival rate at 5 years for BLCA hovers around 70% [29]. In recent times, immunotherapy has gained recognition as a particularly promising approach for treating BLCA, with intravesical immunotherapy viewed as the optimal standard of care for individuals with high-grade, high-risk BLCA, aiming to minimize or prevent recurrence and disease progression [30]. Nonetheless, the expenses associated with these treatments can be high, and their effectiveness can differ widely among patients [31]. The variation observed in BLCA can lead to diverse clinical outcomes and degrees of tumor aggressiveness, underscoring the significance of phenotypic characteristics and their impact on prognosis as well as treatment alternatives.

Cellular senescence plays a complex role in BLCA, influencing both the advancement and inhibition of tumors. This process is marked by an irreversible halt in the cell cycle, serving as an effective mechanism to restrict the growth of damaged or potentially cancerous cells. In the context of BLCA, cellular senescence can act as a defensive mechanism against tumorigenesis by limiting the expansion of cells harboring oncogenic mutations. Nevertheless, the interplay between cellular senescence and BLCA is intricate. While senescence has the capacity to impede tumor development, there is research indicating that senescent cells may develop a SASP, which might facilitate tumor progression and malignancy. The SASP is characterized by the release of various proinflammatory cytokines and growth factors that can modify the tumor microenvironment, thereby creating conditions that promote tumor progression. For instance, senescent cells can release factors that draw immune cells to the area, which may initially aid in tumor suppression but can eventually lead to malignancy through ongoing inflammation and the activation of growth in neighboring nonsenescent cells [32–34]. In the context of BLCA specifically, recent studies have indicated several mechanisms by which cellular senescence influences tumor characteristics. The accumulation of senescent cells can lead to a state where tumor cell proliferation is halted; however, if these cells persist, they can create a chronic inflammatory environment that supports tumor progression via SASP [35, 36]. Moreover, the expression patterns of senescence-related genes can serve as potential biomarkers for risk stratification and prognosis in BLCA. For instance, research has identified important genes associated with senescence that could be utilized for creating prognostic models, thus enhancing the accuracy of clinical outcome predictions and guiding treatment approaches. Evidence from these models indicates that patients exhibiting increased activity in certain senescence-related pathways may experience poorer overall survival rates [32], indicating the critical interplay between senescence and cancer progression. In addition to its role in tumor growth, cellular senescence is also associated with the immune response in BLCA. Senescent tumor cells can influence the immune landscape significantly. Some studies suggest that nonmalignant senescent cells can elicit a response from immune cells, enhancing immunological surveillance. However, this response is context-dependent and can lead to a paradox where rather than suppressing tumorigenesis, the presence of senescent cells might contribute to tumor promotion due to their SASP factors, which can modulate immune cell activity in a way that favors tumor growth. In conclusion, cellular senescence plays a complicated yet significant role in both the occurrence and development of BLCA. While it can suppress tumor growth initially by preventing the proliferation of damaged cells, it also harbors the potential to contribute to malignancy through its influence on the tumor microenvironment via SASP. Understanding this duality is crucial for developing therapeutics that harness the antitumor effects of senescence while mitigating its potentially harmful consequences.

Our research revealed that PAQR4 functions as an important senescence gene in BLCA, a finding that has not been documented in existing literature. Prior research indicates that PAQR4 may serve as a promising therapeutic target for various tumors, including ovarian cancer, prostate cancer, and renal papillary cell carcinoma [37–39]. Our results indicate that PAQR4 may serve as a prognostic indicator for patients with BLCA and that reducing PAQR4 expression can impede the growth and metastatic potential of BLCA cells.

Despite the novel insights provided by our study, several limitations warrant acknowledgment. First, although we integrated two independent cohorts (TCGA-BLCA and GSE13507), the sample size of normal tissues (19 in TCGA, 9 in GSE13507) remains relatively small, which may limit the robustness of senescence-related gene identification. Second, while our diagnostic model achieved high accuracy (AUC = 0.976), its prognostic performance was moderate (AUC = 0.758). This indicates that the model may require further refinement with larger multicenter cohorts to enhance its generalizability across diverse populations. Third, the experimental validation focused solely on in vitro assays. Future work should include in vivo models to confirm PAQR4's role in tumorigenesis and metastasis, as well as clinical samples to validate its association with immune evasion. Additionally, mechanistic studies exploring how PAQR4 regulates the p53 pathway and SASP factors in BLCA are necessary to elucidate its dual role in senescence and immunosuppression.

## 5. Conclusion

Our extensive research examined how genes associated with cellular senescence contribute to the diagnosis, prognosis, and immunotherapy of BLCA. We discovered PAQR4 as a potential new biomarker for BLCA, which could serve as a fresh target for clinical intervention.

## Data Availability

The data that support the findings of this study are available from the corresponding author upon reasonable request.
